# The Role, Involvement and Function(s) of Interleukin-35 and Interleukin-37 in Disease Pathogenesis

**DOI:** 10.3390/ijms19041149

**Published:** 2018-04-11

**Authors:** Ramatu Omenesa Bello, Voon Kin Chin, Mohammad Faruq Abd Rachman Isnadi, Roslaini Abd Majid, Maizaton Atmadini Abdullah, Tze Yan Lee, Zainul Amiruddin Zakaria, Mohd Khairi Hussain, Rusliza Basir

**Affiliations:** 1 Department of Human Anatomy, Faculty of Medicine and Health Sciences, University Putra Malaysia, 43400 Serdang, Selangor, Malaysia; ramatu42@yahoo.com (R.O.B); mohdfaruqar@gmail.com (M.F.A.R.I.); 2 Department of Medical Microbiology and Parasitology, Faculty of Medicine and Health Sciences, University Putra Malaysia, 43400 Serdang, Selangor, Malaysia; cvk717@gmail.com (V.K.C.); roslaini@upm.edu.my (R.A.M.); 3 International Institute of Management & Technology, Wisma TLT, 51 & 51A, Jalan Sultan Azlan Shah, Titiwangsa Sentral, 51200 Kuala Lumpur, Malaysia; 4 Department of Pathology, Faculty of Medicine and Health Sciences, University Putra Malaysia, 43400 Serdang, Selangor, Malaysia; maizaton@upm.edu.my (M.A.A.); tzeyan.lee@gmail.com (T.Y.L.); 5 School of Foundation Studies, Perdana University, 43400 Serdang, Selangor, Malaysia; 6 Department of Biomedical Sciences, Faculty of Medicine and Health Sciences, University Putra Malaysia, 43400 Serdang, Selangor, Malaysia; zaz@upm.edu.my (Z.A.Z.); khairi@upm.edu.my (M.K.H.)

**Keywords:** cytokines, interleukin-35, interleukin-37, pathogenesis

## Abstract

The recently identified cytokines—interleukin (IL)-35 and interleukin (IL)-37—have been described for their anti-inflammatory and immune-modulating actions in numerous inflammatory diseases, auto-immune disorders, malignancies, infectious diseases and sepsis. Either cytokine has been reported to be reduced and in some cases elevated and consequently contributed towards disease pathogenesis. In view of the recent advances in utilizing cytokine profiles for the development of biological macromolecules, beneficial in the management of certain intractable immune-mediated disorders, these recently characterized cytokines (IL-35 and IL-37) offer potential as reasonable targets for the discovery of novel immune-modulating anti-inflammatory therapies. A detailed comprehension of their sophisticated regulatory mechanisms and patterns of expression may provide unique opportunities for clinical application as highly selective and target specific therapeutic agents. This review seeks to summarize the recent advancements in discerning the dynamics, mechanisms, immunoregulatory and anti-inflammatory actions of IL-35 and IL-37 as they relate to disease pathogenesis.

## 1. Introduction

Under the circumstances of host pathology and defense, the cells comprising the immune system produce an assortment of biological products that enable them to communicate and mount specific and effective immune responses. Among these biological products, cytokines are perceived as crucial mediators, which are integral to the progression and outcome of numerous infections, auto-reactive disorders, allergic illnesses as well as neoplasms in which a range of cytokines are produced to modulate subsequent cellular responses of the host towards the invading pathogen or noxious stimuli. [1].

Essentially, cytokines represent an integrated network of cellular mediators capable of eliciting diverse biological effects influenced by the prevailing state of the organism [2]. They represent a distinct, heterogeneous array of proteins which are fundamental in conveying signals amidst various cells of the immune system and consequently oversee gene expression in these cells; operate the scope and extent of an inflammatory response, in addition to controlling cellular differentiation, expansion, antibody secretion, inflammatory immune responses as well as immune pathology [3,4]. Basically, cytokines mediate the turnout of an effective immune response and serve as an interface between the two arms (i.e., innate and adaptive elements) of an otherwise complex immune system [5].

Usually categorized as being either pro-inflammatory or anti-inflammatory, cytokines are secreted by diverse cell populations upon stimulation [6]. Notably, both pro-inflammatory and anti-inflammatory types elicit distinct responses to immunogens at different stages of an infection. Moreover, cytokine response profiles generated towards the same stimuli have been shown to differ between different individuals, among species and depend on the nature of a disease, either chronic or acute, pathogenic or autoreactive in etiology [7]. Certain cytokines have pleiotropic functions and hence exhibit functional redundancy [8]. Cytokine repertoires influence the make-up of cellular infiltrate(s), cellular activation and intrinsic responses to inflammation. Furthermore, their actions may be “autocrine,” whereby they exert their biological function on the cell(s) that secrete them or “pancrine” in which case they evoke actions on cells other than those from which they are secreted [1].

**Figure 1:**
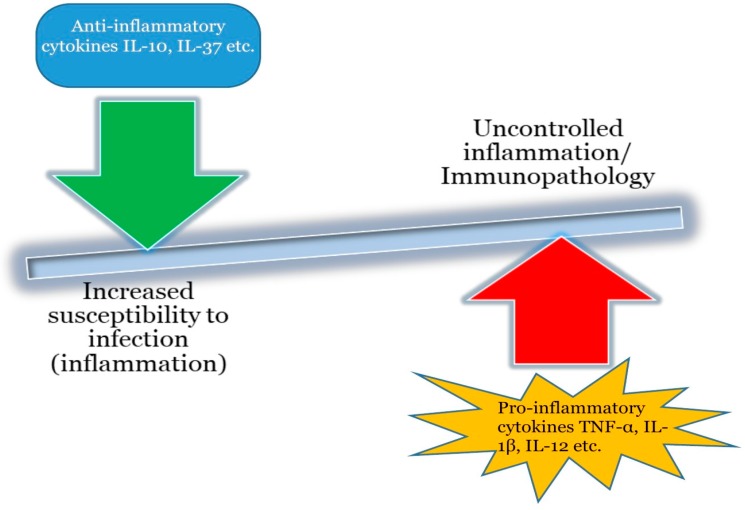
Schematic representation of immunopathology resulting from unregulated cytokine activation.

Although cytokines are beneficial in the initiation and coordination of immune responses, unregulated cytokine signaling inadvertently constitutes principal determinants of immunopathology, giving rise to autoimmune disorders such as rheumatoid arthritis as well as hypersensitivity reactions [9,10]. Cytokines are of central importance in the development of immunopathological symptoms in consort with several diseases owing more or less to the fact that their signaling is complex and tightly regulated and they are capable of acting synergistically, hence, minor disturbances in their homeostasis may result in immunopathology. Figure 1 depicts the consequences of unregulated cytokine activation which will eventually leads to immunopathological events seen in various diseases.

Contemporary advancements in understanding cytokine biology have brought to light the benefits of focusing on this class of proteins as druggable targets for the development of therapeutic biological macromolecules that are efficacious in disease amelioration and highly selective with fewer incidences of ambiguous side effects [1]. Remarkable success has been achieved in the areas of targeting (blocking or enhancing) cytokine activities for therapeutic purposes [9,11]. Over the past decade, IL-35 and IL-37 have been the focus of immunoregulatory cytokine research in a fair share of disease models. Intense investigations into the possible implications of their immune suppressing properties as well as their potential for therapeutic application as cutting-edge biological macromolecule is being explored in diseases ranging from autoimmune diseases, various types of cancer as well as infectious diseases [12].

## 2. Immunobiological Significance of IL-35 in Disease Pathogenesis

**Figure 2:**
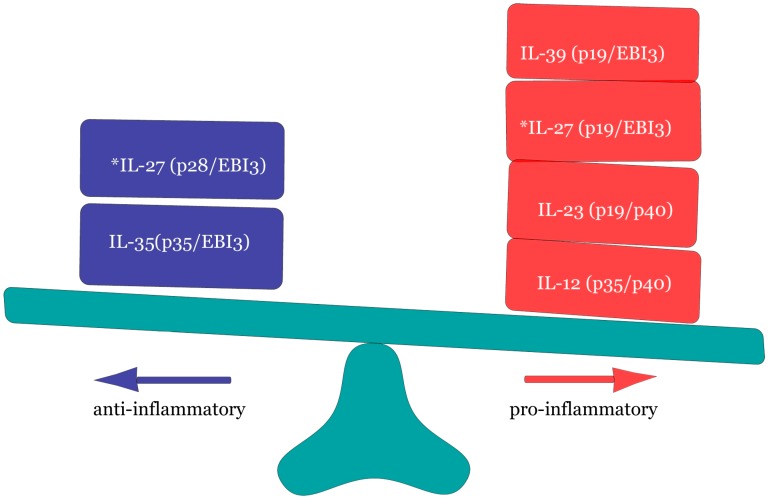
Schematic representation of pro-inflammatory and anti-inflammatory cytokines constituting the IL-12 cytokine family. All family members are made up of an alpha and beta sub unit (α/β) fused together via disulphide bonds. Sensu stricto, IL-12 and IL-23 represent pro-inflammatory members of this family, while *IL-35* is strictly anti-inflammatory. IL-27 is pleotropic in nature. IL-39 on the other hand is relatively less characterized but accumulating evidence point at its pro-inflammatory actions [13].

The IL-12 cytokine family to which IL-35 belongs (as the most recently identified member) is quite unique in that the members comprise of two distinct units; an α-subunit (p19, p28 or p35) along with a β-subunit (p40 or Ebi3). This family of cytokine exists as heterodimeric molecules [12,13], viz IL-12 (p35/p40), IL-23 (p19/p40), IL-27 (p28/Ebi3), IL-35 (p35/Ebi3), IL-39 (p19/Ebi3) as well as IL-Y (p28/p40). Due to similarities in chain pairing, family members exhibit structural homology and feature similar receptors (signaling pathways) for the execution of their respective contrasting biological functions [14]. This family of cytokines exerts multiple functions to produce diverse phenotypic traits. Egwuagu et al. [15] demonstrated their profound actions in shaping and regulating host immunity. These cytokines operate a diverse multiplicity of immune responses, from inflammation promoting T_H_1, T_H_2 and T_H_17 type responses to retroactive anti-inflammatory immune defenses mediated primarily via regulatory T cell (T_regs_) proliferation and expansion of IL-10 expressing T_regs_ [15]. Figure 2 depicts the IL-12 family members and their associated subunits together with their roles as pro-inflammatory and anti-inflammatory cytokines.

Contrasting in expression pattern and biological activity, the serendipitous finding of IL-35 occurred when the α-subunit of IL-12 (p35) was co-incubated with Ebi3 (which is itself a subunit shared by the IL-6 cytokine super family as well as the IL-12 cytokine family) in the pursuit to discover probable catenation pairs for Ebi3 [16]. The ensuing novel heterodimer formed (Ebi3/p35) was later discovered to be capable of exerting potent immunoregulatory actions [16,17]. Both α and β-subunits of IL-35 are largely derived from regulatory Foxp3^+^ T_regs_ distinctive from most IL-12 cytokine family members which are chiefly expressed on the surface of activated dendritic cells, B-cells, monocytes and macrophages viz antigen presenting cells via which they drive T_H_1 responses solely or synergistically in addition to promoting interferon-γ (IFN-γ) generation by T cells [16,18]. Contrariwise, a study by Li et al. [19] involving gene-expression profiles of *IL-35* revealed a broader tissue distribution than was earlier portrayed i.e., confined to T_regs_ [19]. The p35/p40 heterodimer (i.e., IL-12) along with p19/p40 (viz IL-23) constitute the strictly pro-inflammatory members of this enigmatic cytokine family, whereas IL-27 on the other hand is described as capable of exerting pleomorphic actions, specifically driving T_H_1 responses similar to IL-12 on one hand and promoting IL-10 dependent immunosuppression on the other [13,20,21].

IL-35 represents a relatively newcomer to the already established class of extensively characterized suppressive cytokines comprising of IL-10, transforming growth factor-β (TGF-β) as well as the pleomorphic co-IL-12 cytokine family member IL-27 [12]. These suppressive cytokines comprise critical immunomodulatory networks and tolerance-promoting channels essential for adequate functioning of the immune system [12]. Collison et al. [16] characterized IL-35 as a strictly immunosuppressive cytokine possessing the capacity to abolish T_eff_ cell expansion while simultaneously mediating the conversion of naive T cells toward a robust population of induced T_regs_ (iTr) that function exclusively via IL-35 [16,22].

Resting/non-stimulated mouse T_regs_ have been well-established to be capable of secreting IL-35 whereas, non-stimulated human T_regs_ are not capable of doing so [16]. This insinuates that IL-35 is inducible and not fundamentally expressed by humans. This was further substantiated by Li et al. [19] who demonstrated the presence of *IL-35* mRNA in monocytes, smooth muscle cells as well as endothelial cells only after pyrogenic perturbation with lipopolysaccharide (LPS) or inflammatory cytokine(s). Taken together, this data delineates the role of IL-35 as a responsive cytokine which is only secreted in reaction to inflammatory stimuli [19]. Contrarily a later study by Mao et al. established that IL-35 was intrinsically expressed in human placental trophoblast cells [23].

Notably, endothelial cells, activated B cells, dendritic cells, monocytes, smooth muscle cells as well as macrophages have been reported to secrete IL-35 although to a lesser extent [24,25]. Secreted in response to interferon-γ (IFN-γ), IL-35 acts as an agonist on toll-like receptors (TLR) 3 and 4. The immunoregulatory function of IL-35 occurs principally via the inhibition of CD4^+^ effector cell expansion (T_H_1 and T_H_17 included), along with the enhancement of T_reg_ cell proliferation and IL-10 production [24]. IL-35 functions by expanding T_reg_ cell population effectively inhibiting T_eff_ cells thereby forestalling immune injury in the event of an ensuing chronic infection and inhibition of T_H_17 cell differentiation away from unbridled autoimmune reaction [24].

Although described as an initiator and effector of anti-inflammatory signaling, the principal actions of IL-35 in living systems are yet to be clearly elucidated [26]. It is however evident that in the presence of an antigen presenting cell-free culture, IL-35 directly suppresses the proliferation of T_eff_ cells. Additionally, IL-35 deficient T_reg_ cells have been observed to display greatly diminished suppressive ability in vivo resulting in a failure to hinder the homeostatic expansion of T_eff_ cells and predisposition to subsequent exuberant autoimmune sequelae, evidenced by the impaired in vivo ability of IL-35 deficient T_regs_ to alleviate colitis [16].

**Figure 3:**
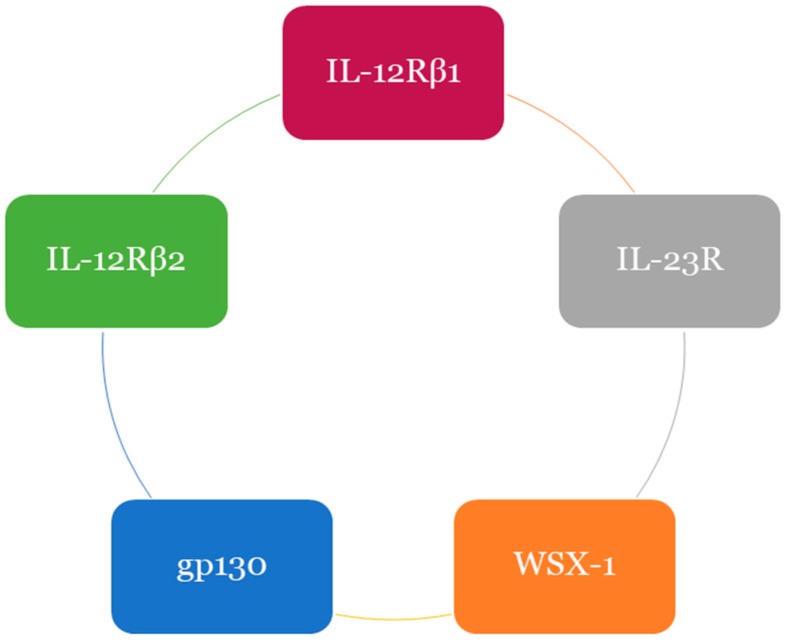
The receptor subtypes that make up the IL-12 cytokine receptor family, comprising five receptor sub units. A propensity for receptor sharing among family members exists owing to similar α and β subunits within this cytokine family. Bipartite receptors pairs (homodimers or heterodimers are employed by IL-35 for signal transduction).

The IL-12 receptor (IL-12R) family comprises of five main receptor subunits. It is presumed that a combination of receptor types is used by IL-35 for signal transduction. Similarly, various unique receptor chain combinations are employed by other family members for signal transduction including interleukin-12 receptor (IL-12Rβ1 and IL-12Rβ2), interleukin-23 receptor (IL-23R), interleukin 27 receptor (WSX-1) and glycoprotein 130 receptor (gp130) as illustrated in Figure 3 [27].

Interestingly, IL-35 does not comply with the quintessential high affinity and lower affinity receptor complex paradigm signaling peculiar to a majority of cytokines. The signaling pathway for IL-35 appears to overlap with that of IL-12 as well as IL-27. This is evidenced by the deployment of signals via its non-preferential binding to homodimers (“IL-12Rβ2: IL-12Rβ2” and “gp130:gp130”) or heterodimers of IL-12Rβ2 as well as gp130 which comprise of IL-12R and IL-27 receptor (viz “gp130: IL-12Rβ2” and “IL-12Rβ2: WSX-1”) respectively [28]. gp130 receptor is ubiquitous in addition to being an integral moiety of receptors for numerous cytokines including; IL-6, IL-11 and IL-27. The IL-12Rβ2 on the other hand is strictly an IL-12 cytokine family receptor [29]. Signal transduction of IL-35 is initiated upon its binding to either homodimers or heterodimers comprising of gp130 and/or IL-12Rβ2. Following receptor engagement, signal transduction takes place via the janus kinase and signal transducer and activator of transcription (JAK-STAT) pathway [28]. Phosphorylation of signal transducer and activator of transcription STAT1 was demonstrated to occur upon binding of IL-35 to gp130 receptor whereas STAT4 phosphorylation occurs upon binding of IL-35 to IL-12Rβ2 [28]. Co-incubation of IL-35 with T cells deficient in either STAT1 or STAT4 revealed a marked reduction in their suppressive capacity, hence, the coordinated role of STAT1 and STAT4 are essential for IL-35 mediated suppression [28].

Collison et al. [28] illustrated that signaling via homodimer receptor pairs is moderated by STAT1 and STAT4 with a resultant partial loss of IL-35’s suppressive activity when compared to its signaling via the wholly functional IL-12Rβ2-gp130 heterodimeric receptor complex [30]. Signal transduction via homodimer receptors culminates in the negation of T_eff_ cell expansion while failing to induce their conversion to iTr35, reason being that only one arm of signal transduction pathway (comprising the homodimer) is “switched on”. Contrastingly, Wu et al. [31], reported that signal transduction via the homodimer receptor pair comprising IL-12Rβ2:gp130 is capable of mediating both iTr35 induction as well as T_eff_ cell suppression. Evidence also shows that IL-12Rβ2 can be expressed by both B cells and dendritic cells invariably influencing the bioactivity of IL-35 in the immune system [32]. Research by Shen et al. [33] illustrated that signal transduction of IL-35 in B-cells is moderated via a heterodimer receptor complex comprising of IL-12Rβ2: WSX-1.

### 2.1. IL-35 Generates a Lineage of Functionally Suppressive IL-35 Expressing T-cells (iTr35) in Human and Murine Subjects

The proliferation of induced T_regs_ cells (iTr) in the periphery from naive CD4^+^ T cells constitutes a substantial research interest in the context of their probable therapeutic application as tolerance-promoting immunobiologicals [34]. This is further supported by the fact that iTr cells can be produced in copious numbers hence making them ideal therapeutic targets. In addition to the potent inhibitory actions of iTr aimed at specific antigens, these cells have been proven to be beneficial in restoring regulatory networks upon application in conditions where T_regs_ are exhausted or inherently defective [34]. 

**Figure 4:**
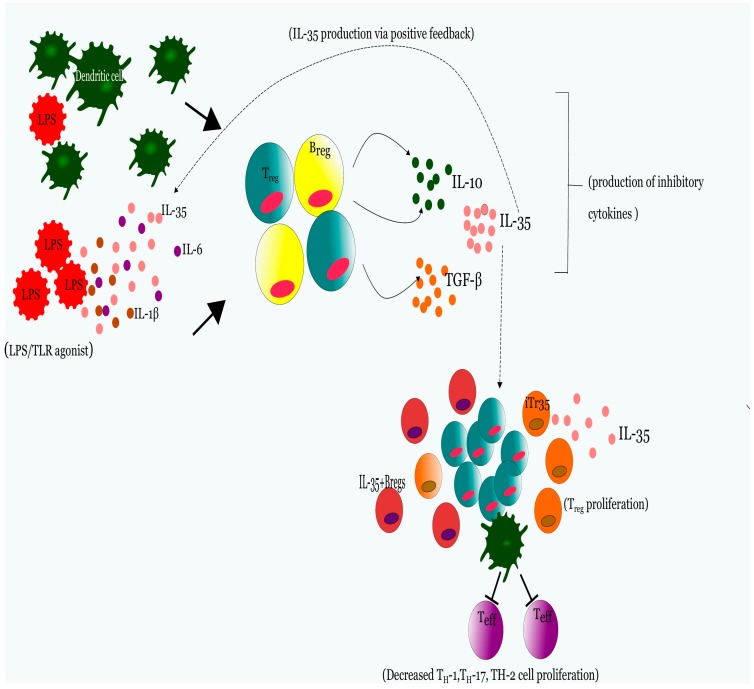
Schematic representation of IL-35 mediated suppression involving the subversion of T_eff_ cell proliferation and the induction of a potent regulatory population of IL-35 producing T_regs_ and B_regs_ that function exclusively via IL-35.

The immunosuppressive attribute of IL-35 is not simply limited to the subversion of T_eff_ cell proliferation along with their ensuing cellular responses; IL-35 can in turn stimulate induction of T_reg_ cells invariably prompting tolerance effect in an infectious state. Earlier research demonstrated that co-incubation of supernatant obtained from IL-35 transfected human embryonic kidney cell line (HEK293T) on naïve mouse or human T_regs_ induced a population of T cells denoted iTr35 cells that were themselves capable of exerting suppressive effects strictly mediated by IL-35, not IL-10 nor TGF-β and in turn these cells were able to enhance immunosuppression by further secreting additional IL-35 [22,35]. Figure 4 depicts the schematic representation of IL-35 mediated suppression.

### 2.2. Function(s) of IL-35 in Autoimmune Disorders

Autoimmune disorders typically hold significance as a leading non-communicable disease worldwide. Despite limited knowledge pertaining to their causative mechanisms, researchers have revealed that these disorders are not strictly governed by a single immunologic anomaly (e.g., generation of bad clones or autoantibodies) but rather span across multiple factors and a range of mechanisms including; a breakdown in tolerance and subsequent auto-antibody release at different sites in addition to generation and selection of bad clones amongst other factors [36]. These disorders are now known to stem from the intricate interplay between diverse cell populations in the immune system (T cells, B cells, classical antigen presenting cells, etc.) culminating in auto-aggressive responses to self viz loss of tolerance [37]. Cytokines represent key elements that operate the ensuing interplay between cells of the immune system and have been documented in some instances to support the recruitment, protraction and multiplication of auto reactive immune cells [36].

Several experimental models have demonstrated the functional profile and regulatory mechanisms of inflammation mitigating cytokines (IL-10, TGF-β) including IL-35 in a range of autoimmune diseases. In broad terms, the suppressive capacity of T_reg_ cells in (*IL-35* deficient) *p35^−/−^* or *Ebi3*^−/−^ murine subjects (achieved via targeted deletion of either of the two genes) is significantly reduced in contrast to their wild-type counterparts [24].

#### 2.2.1. Rheumatoid Arthritis

Typically, rheumatoid arthritis (RA) patients elaborate markedly diminished IL-35 and T_reg_ titers, which by extension hinders their ability to effectively suppress pro-inflammatory cytokine release by T_eff_ cells [6,35]. Niedbala et al. [35] demonstrated a delay in normal disease progression as well as amelioration of disease severity in a rodent model of collagen induced arthritis (CIA), following systematic administration of IL-35 [35,38]. Murine arthritic subjects receiving exogenous IL-35 therapy demonstrated considerable reduction in critical clinicopathological features associated with arthritis (including arthritic paw count, adjacent cartilage and bone erosion, synovial hyperplasia) as well as decreased histological articular damage when compared to their positive control counterparts receiving only phosphate buffered saline [35]. The observed amelioration of CIA by IL-35 was attributed to an IL-35 mediated enhancement of T_regs_ cell proliferation in addition to its restriction of T_eff_ cell expansion and differentiation towards a T_H_1 and T_H_17 phenotype [39]. Consequently, IL-35 augmented mice elaborated a marked decrease in T_H_1 as well as T_H_17 cytokine titers and high circulating levels of IL-10 in their serum. A study involving human arthritic patients detected lower circulating indices of IL-35 in sera of arthritic patients in comparison to controls [6].

Vascular endothelial growth factor (VEGF) and angiopoietins (Angs) represent key factors which act synergistically to foster synovial angiogenesis and inflammation in RA [40]. The suppressive capacity of varying concentrations of IL-35 on VEGF/Ang2 pro-angiogenic pathway (translated in terms of decreased endothelial cell adhesion, migration and tube formation) was demonstrated in in vitro cultures of human umbilical vein endothelial cells (HUVECs) and an ex vivo RA synovial tissue explant set up [41]. IL-35 was observed to antagonize intrinsic as well as VEGF-induced tube formation in vitro and in vivo in addition to the observed inhibition of HUVECs migration and adhesion in vitro. The observed antiangiogenic property of IL-35 was surmised to occur as a result of its direct inhibitory action on Ang2 expression in addition to an IL-35 mediated interruption of signal transduction via the angiopoietin receptor (Tie2) pathway. Furthermore, the enhanced secretion of vascularization/inflammation response modulating genes *matrix metalloproteinase 2* (*MMP-2*) and *matrix metalloproteinase 9* (*MMP-9*) in addition to increased IL-6 and IL-8 secretion upon exogenous VEGF and/or Ang2 application to HUVECs and RA synovial explants was potently abrogated by IL-35 [41].

#### 2.2.2. Experimental Autoimmune Encephalomyelitis (EAE)

Experimental autoimmune encephalomyelitis (EAE) represents a murine (experimental) archetype for the human condition multiple sclerosis [42]. Conventionally, EAE is initiated by the generation of pathogenic T helper cells in the central nervous system (CNS) via immunization of myelin oligodendrocyte glycoprotein (MOG) to susceptible mice causing CNS demyelination (evidenced by neuroinflammation and limb paralysis). Susceptible mice revealed a complete protection from CNS symptoms upon iTr35 treatment as compared to *Ebi3*^−^/^−^ mice treated with iTr35 in which disease progression was indistinguishable from their saline treated counterparts. IL-35^+^ B_regs_ generated from IL-35 releasing B lymphocytes also accounted for disease amelioration [33]. Shen et al. [33] demonstrated that IL-10 is required by B_reg_ cells in addition to IL-35 for the effective resolution of EAE, reason being that a deficiency in either one of the two cytokines abrogated completely the regulatory potential of B cells. Experimental autoimmune uveitis (EAU) serves as a template for preclinical research on human uveitis (or eye-specific antigens). EAU is potently suppressed by IL-35, this is evidenced by reduction in ocular inflammation and associated disease severity upon recombinant IL-35 therapy (rIL-35). Such potent disease suppression is thought to be mediated by the inhibitory actions of IL-35 on T_eff_ cell proliferation in addition to its suppressive actions on T_H_17 cell expansion as well as the enhancement of IL-35^+^ B_regs_ and T_reg_ cell expansion [32]. 

#### 2.2.3. Hashimoto’s Thyroiditis

A role was delineated for IL-35 in Hashimoto’s thyroiditis—an autoimmune thyroid disorder (AITD) that targets the thyroid gland, characterized by defective (self)-tolerance to thyroid antigens. Clinical symptoms develop as a result of the attacks on the thyroid gland by auto antibodies released defectively by a malfunctioning immune system. The disease features decreased thyroid follicular cell viability resulting from inept expression of apoptosis pathway molecules and genes, *Fas or B-cell lymphoma-2* gene (*Fas* or *Bcl-2*) respectively, lymphocytic thyroid infiltration and thyroid hyperplasia. Disease initiation and progression has been attributed to defective T_regs_ cell function and expansion, in addition to a concomitant increase in follicular helper T cell activation [43]. Yilmaz et al. [44], illustrated that IL-35 serum concentrations correlated inversely with the levels of thyroid stimulating hormone (TSH) and anti-thyroid peroxidase antibodies (TPOAb). They proposed that lower circulating levels of IL-35 (which hinders T_reg_ proliferation and function) promotes a loss of tolerance owing to a decrease or functional impairment of T_regs_ invariably setting up a favorable environment for the development and exacerbation of autoimmune disorders including Hashimoto’s thyroiditis [44]. Owing to the previously cited suppressive action of IL-35 on mouse B_regs_, decreased IL-35 titers were surmised to be a predisposing factor to a B_reg_-mediated increase in TPOAb expression [32]. Consequently, it was deduced that optimal IL-35 titers are essential for protection against thyroid immunopathology and this action of IL-35 is mediated through its action on T_reg_ cell expansion rather than its direct abrogation of pro-inflammatory cytokine function [44].

#### 2.2.4. Multiple Low Dose Streptozotocin (MLDSTZ)

Multiple low dose streptozotocin (MLDSTZ) represents a murine model of human diabetes. Susceptible mice, pre-treated with rIL-35 and subsequently subjected to MLDSTZ treatments, failed to develop hyperglycemia and stayed normoglycaemic in contrast to their saline-treated age and sex-matched controls, where insulitis and hyperglycemia were manifested [45]. The proffered mechanism of disease attenuation was described as an IL-35 mediated build-up in anti-inflammatory signaling particularly, IL-10 and IL-35 [45]. Additionally, IL-35 was proffered to effectively reverse the phenotypic shift of (functionally impaired) T_regs_ restoring their suppressive capacity [45].

Li et al. [46] demonstrated that sera from patients with active stage irritable bowel disease (IBD) had notably lower levels of IL-35 compared to healthy controls. Previous studies report a decrease in T_reg_ cell count to be associated with ulcerative colitis. Suffice to say that T_regs_ being a primary source of IL-35, such decrease translates to a corresponding decrease in peripheral circulating levels of IL-35 invariably offsetting an imbalance in the mitigating counter-inflammatory response required to effectively curb mucosal release of inflammatory mediators consequently exacerbating symptoms in patients with IBD [46]. Conversely elevations in the level of IL-35 was detected in T_reg_ and B_reg_ cells of IBD patients further supporting the immunoregulatory function of IL-35 [47].

### 2.3. Role of IL-35 in Respiratory Disorders

Earlier research accorded primacy to polymorphisms in cytokine genes and their signaling pathways in the pathogenesis of (allergic) airway disorders such as asthma [48]. Both T_H_2 and T_H_17 cells have been implicated as the principal effector cells involved in asthma pathogenesis. T_H_2 have been reported to mediate local (airway) recruitment of inflammatory cell subsets (mast cells, eosinophils, etc.) while T_H_17 cells represent the principal source of neutrophil attracting cytokines in the lung parenchyma [48].

Kanai et al. [49], ascribed a central role to IL-35 in an experimental model of asthma. Excessive airway eosinophilia observed in susceptible *Ebi3*^−^/^−^ mice previously sensitized and subsequently exposed (intratracheally) to ovalbumin (OVA) or LPS was potently suppressed upon rIL-35 administration. Furthermore, the associated increased recruitment of eosinophil-attracting chemokines, eotaxin-1 and eotaxin-2 (CCL11 and CCL24) as well as ensuing airway eosinophilia were potently antagonized by IL-35. Similarly, in vitro studies involving perturbation of human bronchial epithelial cells (BEAS-2B cells) with tumor necrosis factor alpha (TNF-α) as well as IL-1β revealed a regulatory role for IL-35 modulated by its suppressive action on STAT1 and STAT3 phosphorylation. The latter of which has been described for its role in regulating CCL11 expression [50]. Treatment with rIL-35 was also reported to result in an increase in BEAS-2B associated suppressor of cytokine signaling 3 (SOCS3) expression thereby enhancing the inhibition of gp130-mediated IL-6 signaling [49].

A significant alleviation in symptoms of allergic rhinitis (nasal rubbing gestures as well as total number of sneezes) was observed upon rIL35 administration in a murine model of allergic rhinitis [51]. Intranasal IL-35 administration was reported to negate T_eff_ cell responses as well as the pathological production of IL-4 and IL-5 (T_H_2 cytokines) both implicated in the exacerbation of allergic rhinitis. Furthermore, an IL-35 mediated increase in circulating IL-10 expression was observed, which in turn promotes T_reg_ survival [51]. 

An earlier study by Chen et al. [52] delineated CD8^+^ T cells as the principal mediators driving the lung and airway infiltrations (i.e., neutrophilia) peculiar to individuals suffering from chronic obstructive pulmonary disease (COPD). Investigation in to the dynamics of IL-35 secretion in asthma and COPD revealed lower IL-35 titers in COPD patients compared to the asthmatic and control group. The observed difference in IL-35 titers (between COPD sufferers and asthmatic patients) was attributed to the disparate cellular sources that promote each airway pathology namely; CD4^+^ T cells in asthma and CD8^+^ T cells in COPD.Foxp3^+^ T_regs_ cells are a principal source of IL-35, hence the higher expression levels in asthma. Moreover, the higher levels of IL-35 observed in disease-free control groups was attributed to inherent (effective) anti-allergy mechanism mounted towards sensitization from exposure to typical everyday environmental allergens. This implies that maladaptive anti-allergy mechanisms may contribute to the lower IL-35 titers observed in respiratory disorders [52].

### 2.4. A Role for IL-35 in Cardiovascular Diseases

Atherosclerosis is a consequence of the interaction between immune effector molecules and metabolic susceptibility which trigger lesions (atheromata) in arterial vasculature [53]. Compelling evidence has delineated the key role of T_H_1 cytokines (particularly IL-1β and IL-18) in aberrant immune responses. These aberrant immune responses foster the propagation and activation of arterial lesions resulting in the development of notoriously unstable atherosclerotic plaques and thrombi. Rupture of (unstable) plaques or arterial tree occlusion by a thrombus leads to cardiovascular events with varying (potentially fatal) clinical outcomes including myocardial infarction, cerebrovascular accident (stroke) as well as coronary artery disease [54].

Sanchez et al. [54], demonstrated the involvement of *IL-35* gene polymorphisms in atherosclerosis and its associated clinical ramifications (e.g., coronary artery disease—CAD). Two polymorphisms in the *IL-35* gene namely; *Ebi3* rs428253 and *IL-12A* rs2243115 were found to be associated with a decreased risk of premature CAD development. The same polymorphisms were found to be associated with reduced risk of type-2-diabetes mellitus and metabolic syndrome respectively, both of which are known cardiovascular disease risk factors. Surprisingly, no association was observed between the two polymorphisms and IL-35 levels, probably due to the fact that IL-35 levels were measured in systemic circulation and not localized to the site of sclerotic lesions [54].

Significantly decreased amounts of IL-35, IL-10 as well as TGF-β were observed in plasma of patients with coronary artery disease (CAD) with a concomitant elevation in plasma IL-12 and IL-27 levels. IL-35 showed a positive correlation with left ventricular ejection fraction in the CAD patients studied hinting at the involvement of IL-35 in CAD and its potential relevance as a prognostic marker for CAD [55].

### 2.5. IL-35 Mediates Inflammation in Sepsis

Systemic inflammation in conjunction with unfettered release of large amounts of pro-inflammatory mediators results in the systemic inflammatory response syndrome (SIRS) peculiar to sepsis [56]. A potentially fatal illness in which signs and symptoms are often ambiguous, sepsis normally arises from aberrant host immune response to severe infection. Sepsis is responsible for a high percentage of mortality in adults and neonates [57]. The physiology of cytokines has been extensively evaluated for their likely relevance as prognostic indicators in patients suffering from sepsis. Previous research utilizing IL-10 and IL-27 (an IL-12 cytokine family member) in murine models of sepsis have yielded promising outcomes directed towards the understanding of cytokine dynamics in septic shock. Hence, IL-35 which is also a member of the IL-12 cytokine family was therefore examined for its role in the pathogenesis of sepsis [57]. 

A study investigating the dynamics of IL-35 in both adult and pediatric septic patients revealed higher circulating levels of IL-35 which were strikingly consistent with the degree of disease severity [58]. IL-35 levels were observed to display a positive correlation with the levels of other pro-inflammatory mediators released in sepsis (including; IL-6, IL-8, IL-27, TNF-α and IL-10). An inquisition on the effect of neutralizing IL-35 via the administration of mouse anti-IL-35p35 antibody, demonstrated an increase in peritoneal neutrophil recruitment as evidenced by their increased numbers in peritoneal lavage fluid of IL-35 neutralized mice in contrast to placebo treated healthy mice. Likewise, higher titers of pro-inflammatory mediators (viz TNF-α etc.) at an early time point (6 h) were observed in anti-IL-35p35 treated mice which implies a preservation of vital early inflammatory cytokine response necessary for typical immune activation and pathogen clearance. Quantitation by polymerase chain reaction (PCR) of organ lysates obtained from septic rodents revealed high levels of IL-35 subunits (p35 and *Ebi3* as early as six hours post disease initiation) particularly in the lungs and spleen. As an added advantage, Du et al. [58] revealed IL-35 was able to predict the likelihood of sepsis onset in neonates in a capacity which was highly comparable (and potentially superior) to procalcitonin a known predictive marker for sepsis. Certain immune cells (dendritic cells, monocytes, macrophages, etc.) have been documented to contribute to the cytokine repertoire in sepsis upon activation. Hence, human and murine elevations in IL-35 concentration with ongoing sepsis were ascribed to the stimulation of immune cells at the onset of infection. Detailed reappraisal of the study outcome implicated IL-35 in the pathogenesis of sepsis as well as its probable beneficial application as a predictive (prognostic) marker for disease outcome in septic patients [58].

### 2.6. Role for IL-35 in Connective Tissue Disorders

#### 2.6.1. Systemic Sclerosis (Scleroderma)

Atypical TGF-β activation of dermal and tissue fibroblasts has been proposed to be the primary insult driving the clinical manifestations seen in systemic sclerosis. This multi system connective tissue disorder is characterized by exaggerated build-up of collagen and extracellular matrix around vital organs ultimately resulting in varying degrees of tissue fibrosis and diverse organ malfunction. Tomcik et al. [59] implicated IL-35 in the pathogenesis of systemic sclerosis. He investigated lesional IL-35 expression in sclerotic dermal sections and reported an upregulation of serum IL-35 levels in systemic sclerosis patients as compared to their control counterparts. Moreover, overexpression of both subunits comprising IL-35 (i.e., p35 and Ebi3) was observed in sclerotic dermal lesions from either localized or disseminated sclerosis patients particularly in inflammatory cells and fibroblasts. Sustained elevations of IL-35 levels were observed upon culture of fibroblasts previously acquired from sclerotic skin lesions. Likewise, TGF-β application to cultured (normal) fibroblasts induced IL-35 expression which in turn promoted a switch of resting fibroblasts to an activated state thereby promoting the release of IL-35 and collagen, inadvertently enhancing abnormal TGF-β signaling via a positive feedback loop [59]. T_regs_ are the main source of IL-35 and their numbers are concurrently increased with increasing IL-35 concentration in systemic sclerotic patients. Studies have reported a positive correlation between T_reg_ expansion and clinicopathological features of systemic sclerosis as well as disease progression owing to a characteristic loss of their suppressive function (phenotypic shift) with a resultant CD4^+^ CD25^+^ Foxp3^low^ CD45RA^−^ (non-suppressive) phenotype and an associated propensity to secrete IL-17 [59].

According to Tomcik et al. [59], capillaroscopic assessments in early stage sclerotic patients demonstrated a negative correlation with IL-35. Early stage sclerotic patients demonstrated higher IL-35 levels when compared to patients with a more chronic (protracted) disease duration. IL-35 is known to signal via STAT1 and STAT4, consequently, the molecular mechanisms of IL-35 in systemic sclerosis were adduced to IL-35 mediated influence of pro-fibrotic STAT4 clones on T cell activation, expansion as well as patterns of cytokine release [59]. Hence, Dantas et al. [60] suggested exploiting the dynamics of IL-35 in systemic sclerosis as a serological marker/prognostic indicator.

#### 2.6.2. Actions of IL-35 in a Murine Model of Systemic Lupus Erythematosus (SLE)

Loss of peripheral and central immune homeostasis (tolerance-promoting B and T cells) in addition to subsequent auto-antibody generation and ensuing organ damage are key features of systemic lupus erythematosus (SLE). A study was undertaken by Cai et al. [61] to characterize the immune-regulatory profile of IL-35 as well as its soluble receptors (gp130 and IL-12Rβ2) from peripheral blood mononuclear cells (PBMC) of SLE patients with varying disease activity indices (SLEDAI). Significantly elevated titers of IL-35 were observed in SLE patients unlike their control counterparts. The authors delineated an inverse correlation between the proportion of peripheral circulating T_regs_ cells and disease activity in SLE patients. Additionally, an increased T_eff_ cell to T_reg_ cells ratio (T_eff_; T_reg_) was observed in patients suffering from severe SLE [61]. This observation is in line with reports from previous studies describing a reduced Foxp3^+^ expression by T_regs_ of SLE patients in conjunction with an intrinsic dysfunction of their suppressive capacity. Taken together the authors [61] surmised that the flawed suppressive capacity of T_regs_ from SLE patients may represent the primary element underpinning the immunopathogenesis of SLE. They further interpreted the induction of IL-35 production as a direct result of stimulation by the inflammatory milieu following T_eff_ cell stimulation and adduced inflammatory pathogenesis of SLE to diminished expression and functional capacity of T_regs_ cells. Despite the elevated levels of IL-35 seen in SLE patients, Cai et al. [61] speculated that the expressed IL-35 was unable to sufficiently induce a robust population of T_regs_ cells owing to the decreased expression of gp130 receptor on the surface of CD4^+^ T-helper cells. Suffice to say, a higher expression of gp130 receptor is pertinent for the actualization of suppressive action by IL-35 [61].

In a murine model of SLE, daily administration of recombinant IL-35 (800 ng rIL-35/mouse) was observed to suppress SLE. Cai et al. [61] opined that rIL-35 restored the (otherwise absent) delicate balance between autoimmune tolerance mediators and inflammatory immune response in SLE murine subjects and ascribed this action to effects of IL-35 on T_eff_ cells (viz abridged expansion and effector function). Recombinant IL-35 brought about a reduced T_H_17 effector cell differentiation from CD4^+^ T_H_ cells, which culminated in propagation of immune tolerance in SLE mice. Additionally, significantly decreased SLE-specific plasma auto-antibody concentrations were observed. The action of IL-35 on SLE-specific auto-antibodies suggests that other regulatory cell populations (e.g., B_reg_ cells) may contribute to the actualization of IL-35 mediated immunosuppression. Tolerance-promoting attribute of IL-35 was linked in part to its expansion of peripheral, thymic and splenic IL-10 producing B_regs_ cells [61].

### 2.7. Actions of IL-35 in Hepatitis Infection

Independent researchers have illustrated the immunomodulatory role of IL-35 in hepatitis C virus infection. Liu et al. [62] observed a direct correlation between hepatitis C viral load and IL-35 concentration. The role of IL-35 was described as being somewhat ambiguously centered around its influence on T cell activity (in a specific and non-specific manner). Although failing to show a correlation with the levels of alanine amino transferase (a liver enzyme usually elevated in hepatic dysfunctions) and liver inflammation, IL-35 mediated a reduction in the levels of HCV-induced upregulation of STAT1 as well as STAT3 signaling. Additionally, IL-35 mediated suppression of pro-inflammatory cytokine production was adduced to an inhibition of STAT signaling by IL-35 in both hepatitis C virus infected cells as well as the hepatocellular carcinoma cell line (Huh. 7.5). There was however no evidence of any direct action of IL-35 on viral replication [62].

A study by Shi et al. [63] aimed to characterize the dynamics of IL-35 expression in patients suffering from chronic severe hepatitis B (CSHB), chronic hepatitis B (CHB), liver cirrhosis (LC) and asymptomatic carriers (ASC). IL-35 titers were elevated in sera of patients with HBV infected compared to normal controls. Essentially a progressive increase in IL-35 serum levels was observed with increasing disease severity (i.e., CSHB > CHB > LC > ASC). In CHB patients aside from an elevation of serum IL-35 titers, a positive correlation was also observed between IL-35 levels and partial liver indices, severe liver inflammation and incidence of necrosis [63]. IL-35 was proffered to function in an immune modulating manner by ensuring a homeostatic balance between the hepatitis B virus and T cells in an effort to curtail liver injury. Hence, no direct influence on hepatitis B viral load in the peripheral circulation was ascribed to IL-35 neither was a role proposed for IL-35-mediated direct inhibition of viral replication. The study proposed that the homeostatic balance between HBV and CD4^+^ CD25^+^ T_reg_ cells create an immunotolerant environment which translates to the chronic disease course known to occur in Hepatitis B virus infection. Thus, on the one hand, IL-35 forestalls liver injury provoked by the hepatitis B virus while inadvertently promoting immune tolerance to the HBV via immunosuppressive T_reg_ cells [63].

### 2.8. Function(s) of IL-35 in Cancer

Different assorted yet ambiguous roles in the tumor microenvironment ranging from tumor-promoting to anti-tumor effects have been attributed IL-35 in various types of cancers. A study by Turnis et al. [64] demonstrated a reduction in tumor enlargement in several murine cancer models as a consequence of T_reg_ specific IL-35 deletion in addition to neutralization with an antibody against IL-35. The authors postulated that T_reg_ cell-derived IL-35 fosters tumor inception accomplished via T_reg_ cell exhaustion which in turn restrains anti-tumor immunity [64]. 

Ordinarily, immune surveillance limits tumor (cancer) development, such that oftentimes, tumors which thrive are poorly immunogenic. Decades of research have revealed that tumors have an uncanny ability to survive via the subversion of host immune anti-tumor responses. Contributory to tumor survival is the genomic instability of precancerous cells resulting in clones of varying (often poor) immunogenicity with an uncanny ability to bypass recognition by immune cells (T cells), thereby impeding tumor-directed immune response. Additionally, conversion of naïve T cells to adaptive T_reg_ cells and their subsequent proliferation represents yet another tumor-mediated mechanism adopted to bypass immune surveillance. Suffice to say that tumors create a suppressive environment via the recruitment of immune subverting factors and suppressive cell populations. 

Nishikawa and Sakaguchi [65] demonstrated enhanced tumor rejection and improved disease outcomes upon T cell depletion which was negated upon T cell augmentation. Peripheral and localized cancer tissues (from B16 melanoma and prostate cancer sufferers) reveal enhanced population of immune suppressing T_reg_ cells in tumor microenvironment as well as IL-35 [65]. Researchers recently revealed an enrichment of IL-35 in tumor-recruited T_reg_ cells where it is adduced to partake in immune evasion. T cell exhaustion which is a hallmark of most cancers has been linked to T_regs_ and their associated inhibitory cytokines. The phenomenon (T cell exhaustion) was reversed by simultaneous blockade of T_regs_, Programmed cell death protein-1, IL-10 or TGF-β signal transduction [65]. 

An earlier study by Wang et al. [66] delineated tumor-promoting property of IL-35 in two different murine models of cancer, namely plasmacytoma J558 and B16.F10 melanoma. In these cancer models, IL-35 was observed to considerably increase tumorigenesis in both immunocompetent mice and their immune deficient counterparts [66]. Cao et al. [67] reported that the observed tumor promoting property associated with IL-35 is mediated via the inhibition of anti-tumor cytotoxic T lymphocyte (CTL) responses which is driven by CD4^+^ CD25^+^ T_regs_ recruitment at malignant sites in addition to the build-up of myeloid derived suppressor cells (MDSC) in the tumor microenvironment [66,67]. Additionally, cancer cell resistance to chemotherapy is shown to be mediated by signaling of IL-35 through its gp130 homodimer receptor which in the interim is one of the receptors upregulated in tumor cells [68]. Thus, it is highly probable that IL-35 signaling via gp130 homodimer receptor promotes cancer cell resistance to CTL destruction [28].

A later study undertaken by Zeng et al. [69] to elucidate the action(s) of IL-35 in human colorectal cancer (CRC) patients revealed elevated levels of IL-35 in sera and cancer cell lysates of CRC sufferers. The levels of IL-35 showed high correlation with the clinical stage of tumor as well as the degree (extent) of malignancy. In addition, appreciable reduction of serum IL-35 was observed in patients after surgical resection of tumors, revealing that poor prognosis in CRC is linked to elevated IL-35 titers. Furthermore, negative expression (depletion) of *Ebi3* gene a subunit of IL-35 correlated with a better prognosis and increased overall survival and disease-free survival in a study undertaken to evaluate IL-35 expression patterns in nasopharyngeal carcinoma cells [70,71].

Contrarily, Long et al. [72] cited that disease progression in hepatocellular cancer patients is associated with a decrease in IL-35 expression by tumor tissues. A recent study by Zhang et al. [73] illustrated a beneficial role for IL-35 in inflammation related tumors. He provided evidence that IL-35 production is reduced in colon cancer in comparison to healthy controls and that applying various concentrations of IL-35 to human colon adenocarcinoma cell line (DLD1) and human colorectal adenocarcinoma cell line (HT-29) suppressed their proliferation (invasiveness and migration) in vitro. Additionally, IL-35 was shown to decrease the expression of β-catenin (a molecule known to be promote colon cancer development) invariably hindering colon cancer progression as well as sensitizing colon cancer cells to chemotherapeutic agents [73]. Increased IL-35 titers in a cohort of patients suffering from papillary thyroid carcinoma (PTC) with concurrent Hashimoto’s thyroiditis (HT) was surmised to be beneficial due to the observed improved prognosis in this group of patients (PTC + HT) compared to their counterparts with either PTC or nodular goiter [74].

The deployment of small interfering RNA (siRNA) directed towards *Ebi3* in lung cancer cells was shown to impede lung cancer cell proliferation albeit stable *Ebi3* gene expression confers growth promoting activity in vitro and is associated with poor prognosis [70]. Taken together, numerous human cancer tissues produce ample amounts of IL-35 which hints at the possibility of its application as an independent prognostic biomarker for various types of cancers. On the other hand, anti-IL-35 may serve as a potential anti-tumor therapeutic biomolecule in various malignancies [75]. The overall involvement of IL-35 in disease pathogenesis is summarized in Table 1. 

## 3. The Immunobiology and Signaling of IL-37

IL-37 is described as a newly identified member of the IL-1 cytokine family. This class of structurally analogous and predominantly pro-inflammatory cytokines comprises IL-1α, IL-1β, IL-18, IL-33, IL-36α, IL-36β and IL-36γ [76]. Formerly known as IL-1F7, IL-37 is cited as a negative regulator of immunity and a ligand to the α-receptor of IL-18 (IL-18Rα) as well as IL-18 binding protein (IL-18BP). It is secreted as a precursor molecule in the cytoplasm and nucleus [77]. In the cytoplasm, IL-37 is secreted in close proximity to the plasma membrane, golgi apparatus as well as the endoplasmic reticulum as a precursor molecule. The precursor molecule subsequently translocates to the nucleus following cleavage giving rise to a biologically active viz the mature form of IL-37 [78]. 

Owing to its close structural semblance with IL-18 and mediation of its immunoregulatory actions by its binding to IL-18Rα, IL-37 is classed along with IL-1 as well as IL-33 under the IL-18 sub-family. Cited as a potent inhibitor of innate and acquired immunity, monomeric IL-37 has been delineated to be critically important for the moderation of its biological activity by reason of the observed accession in its biological activity following a shift from dimeric to monomeric configuration [78]. Increased bioactivity upon monomerization as is seen with IL-37 is a less commonly observed phenomenon with the IL-1 family of cytokines where multimerization is pivotal and exclusively associated with increased biological activity [79].

### 3.1. Biological Occurrence of IL-37.

Immunohistochemistry investigations shed light on the expression profiles of IL-37 in human tissues. A variety of normal tissue types (human blood monocytes, tissue macrophages, synovial cells, tonsillar B cells, plasma cells, keratinocytes as well as epithelial cells of the kidney and intestine) were found to express IL-37 along with tumors and neoplastic cells [80]. Constitutive IL-37 expression has been reported in PBMC as well as in the cytoplasm of monocytes. As revealed by immunocytochemistry, plasma cells represent the chief cellular source of IL-37 whereas, IL-37 is prominently expressed in the cytoplasm of monocytes [80].

Alternative splicing of the *IL-37* gene in humans creates five (5) variant isoforms bearing variable tissue specific expression patterns along with slight differences in their amino acid sequences. IL-37b is the longest and most elaborately characterized of all the IL-37 isoforms and it bears the most complete set of exons [78,80]. Comprising of precursor and mature forms, (both of which are biologically active), mature IL-37 binds more readily to the receptor when compared to its precursor [78,81]. Production of IL-37a is confined to the brain and the presence of IL-37b has only been confirmed in the kidney, while the heart, bone marrow and testis have been reported to elaborate IL-37c, IL-37d and IL-37e respectively [78,80,81,82,83]. IL-37b has significant amino acid homology with IL-18, it represents the biologically functional isoform and is the most studied of all the IL-37 isoforms [78]. Unlike their functional variants, IL-37 c and IL-37 e are speculated to be non-functional owing to their atypical (configuration) folding [84]. 

The *IL-37* gene is noticeably absent in mice, [77,78]. There is yet to be any reports of a functional mouse homologue to IL-37 or genomic sequence comparable to IL-1F7 hence the adoption of transgenic murine for studying the dynamics of this cytokine [77,78,82]. IL-37 has been cited as capable of exerting potent anti-inflammatory actions in addition to suppression of pro-inflammatory cytokine production without affecting anti-inflammatory cytokine signaling. Despite being present at low concentrations in blood circulation, IL-37 is rapidly induced to attain much higher circulating concentrations in response to inflammatory stimuli and IL-1β, agonists of TLR or TGF-β [78]. 

Hitherto, IL-1 family of cytokines comprised exclusively of pro-inflammatory cytokines (IL-1, IL-18, IL-36 and IL-33) in addition to two inflammatory cytokine receptor antagonists (IL-1Ra and IL-36Ra) actively involved in modulating T_H_1 and T_H_17 inflammatory responses [78,85]. Accumulating evidence however revealed IL-37 as a natural modulator of inflammation via a feedback loop to suppress exuberant inflammatory immune reaction [86].

### 3.2. Mechanism of IL-37 Mediated Immunosuppression

IL-37 is initially synthesized in a precursor form, which upon stimulation is processed by caspase-1 to its mature form [87]. Mature IL-37 binds to mothers against decapentaplegic homolog 3 (SMAD3) in the cytosol from where it translocates into the nucleus to exert its biological effect of inhibiting pro-inflammatory cytokine gene transcription [78]. IL-37 is released from the cell due to cellular membrane disruption following necrosis or apoptosis. In the cytoplasm of monocytes and PBMC, an upregulation in the levels of circulating IL-37 protein was brought about by agonists of TLRs [78].

Two binding mechanisms have been identified via which IL-37 exerts its immunosuppressive actions, namely;

(a) Binding to IL-18Rα/single immunoglobulin-g-interleukin-1-related receptor (IL-18Rα/SIGGIR) complex to form a tripartite ligand complex (IL-18Rα/L-37/SIGGIR)
(b) Binding to SMAD3 receptor (SMAD3/IL-37)

The inflammation suppressing action of IL-37 via its ternary ligand receptor complex (IL-37/IL-1R8/IL-18Rα) has been illustrated to function by enhancing STAT3 and phosphatase and tensin homolog (PTEN) activity which in turn polarize antigen presenting cells (viz dendritic cells and macrophages) towards an anti-inflammatory profile in addition to inhibiting pro-inflammatory cytokine production via negatively regulating the nuclear factor κ-light chain enhancer of activated B cells (NF-κB) pathway [78,84]. According to research by the cancer center in University of Colorado, IL-37 exerts its anti-inflammatory actions through regulation of dendritic cells (DCs). IL-37 diminishes the elaboration of major histocompatibility complex (MHC) II and the co-stimulatory molecule CD86 (B7-2) on the surface of dendritic cells [46]. This is supported by previous findings where significant reductions in CD86 and MHC II expression were observed in splenic-derived dendritic cells isolated from IL-37 transgenic (IL-37tg) mice upon LPS stimulation [78]. 

Researchers have proffered that IL-37 promotes the generation and migration of semi-mature dendritic cells to lymph nodes; however, being immature, these dendritic cells fail to create immune response due to antigen presenting defects. This is evidenced by the diminished immune system response in IL-37-augmented mice including lower IL-1β, CD40, IL-12 and IL-6, all of which have been implicated in the generation of viable immune response against invading pathogens. Furthermore, studies involving the isolation and subsequent transfection of dendritic cells harvested from IL-37tg mouse lymph nodes into normal (non-IL-37tg) mice exhibited significantly less antigenic challenge under similar conditions when compared to mice transplanted with wild-type (regular) mouse dendritic cells [78]. It was hence inferred that IL-37 expressing dendritic cells are tolerogenic and consequently failed to provoke an immune response. For this reason, IL-37 is regarded as a regulatory cytokine with tolerance promoting properties directed at self-components in addition to the elimination of pathogens while ensuring the least possible (secondary) damage to the host.

Transgenic mice expressing IL-37 were protected from lipopolysaccharide induced septic shock compared to identically challenged age and sex-matched controls. Milder incidences of hypothermia, hepatitis, dehydration acidosis and other symptoms visible upon LPS challenge were displayed by the IL-37tg mice compared to positive control mice [78].

Li et al. [46], attributed the protective effect of IL-37 to an upregulation of CD4^+^ CD25^+^ T_regs_ and their associated suppressive activity induced by an increase in IL-37 mediated expression of cytotoxic T-lymphocyte-associated protein 4 (CTLA4)/Foxp3 and TGF-β production.

### 3.3. Biological Actions of Endogenous IL-37

Administration of a neutralizing antibody against IL-37 to healthy humans following lipopolysaccharide (LPS) stimulation predisposed to an elevation of certain inflammation-promoting cytokines (namely; IL-6, IL-1β and TNF-α). This “house-keeping” function of endogenously secreted IL-37 was further supported by elevations in TNF-α, IL-1α, IL-1β, IL-6, granulocyte-colony stimulating factor (G-CSF) and granulocyte-macrophage colony stimulating factor (GM-CSF) resulting from the blockade of endogenous IL-37 [7]. Additionally, transfection of human and mouse cell lines with IL-37 evoked a decrease in IL-1β-induced cytokine and signaling kinases in macrophage cell lines. This action of IL-37 has been reported to be mediated via its association with signal transducer and transcriptional modulator (SMAD3) [78,80].

### 3.4. Signal Transduction of IL-37

**Figure 5:**
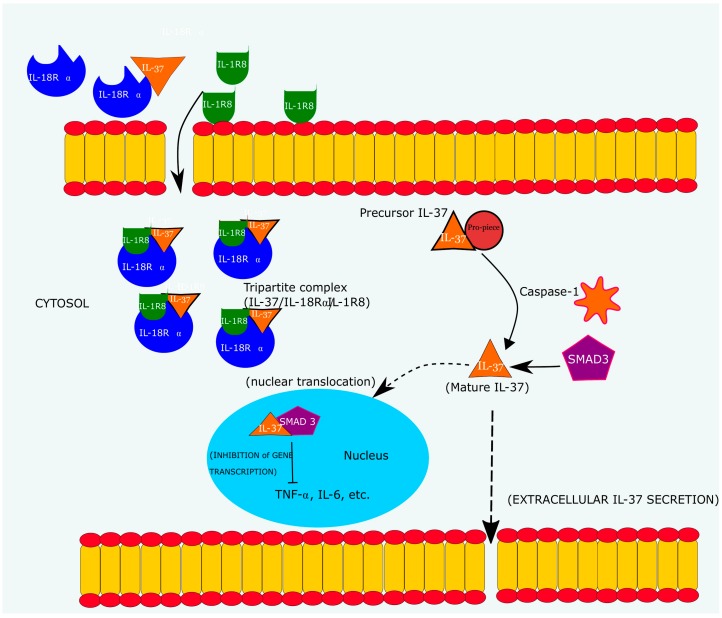
IL-37 processing and release. Precursor IL-37 is processed by caspase-1 to the mature form (IL-37) which subsequently translocates to the nucleus upon binding to SMAD3. The SMAD3/IL-37 complex inhibits pro-inflammatory cytokine gene transcription in the nucleus and in consort with the tripartite molecule (IL-37/IL-18Rα/IL-1R8) inhibits downstream signal transduction pathways including NF-κB and mitogen activated protein kinase (MAPK) pathway. Adapted with modifications from [77].

IL-37 is synthesized as a precursor protein which undergoes cleavage by caspase-1 to a mature IL-37, where it binds to SMAD3 and subsequently translocates to the nucleus where it suppresses pro-inflammatory gene expression as indicated in Figure 5 [77]. IL-37 is capable of exerting both intracellular and extracellular functions [88]. The revelation that IL-37 can be a ligand for IL-18Rα derives from the apparently complex pro-inflammatory manifestations observed upon blocking IL-18Rα in PBMC which endogenously release IL-37 [88]. A predisposition to abrupt inflammatory hyper-responsiveness in mice and cell suspensions following the “silencing” (viz obstruction) of IL-18 receptor α (IL-18Rα) as compared to a milder inflammatory reaction in IL-18 deficient mice affirmed that IL-37 achieves its immunoregulatory function via low affinity non-competitive binding to the IL-18Rα. On that account, IL-37 was consequently deduced to be a ligand for IL-18Rα. Researchers, also posit that IL-37 binds to IL-18BP [83,89].

A critical determinant for extracellular IL-37 anti-inflammatory function could be the recruitment of decoy IL-1R8 viz SIGIRR to the IL-18Rα/IL-37 complex [84]. For inhibitory signal transduction, IL-37 after binding to IL-18Rα recruits SIGGIR which is subsequently activated by the IL-37 precursor protein to provoke a negative signal following its union with IL-18Rα. SIGGIR does not elicit the classical pro-inflammatory signaling cascade that is typical to the IL-1 receptor family, contrariwise, it operates by inhibition of IL-1R and TLR-dependent inflammation [90]. A resultant moderate decrease (~34%) in IL-1β was observed when *SIGGIR* was silenced as compared to a more pronounced reduction (~83%) in IL-37 transfected T_H_1 macrophages possessing intact *SIGGIR* gene [83].

Studies identified an inflammation negating receptor, member of the IL-1 cytokine family IL-1R8 as co-receptor for IL-37 [84]. IL-1R8 knockout mice succumbed to LPS challenge unlike their wild type counterparts. Likewise, SMAD3, a transcriptional regulator in the transforming growth factor-β (TGF-β) network, performs a crucial role in mediating IL-37’s biological effects by assisting in its translocation from the cytoplasm to the nucleus [78].

## 4. Immunoregulatory Mechanisms of IL-37 in Disease Pathogenesis

### 4.1. Actions of IL-37 in Murine (Experimental) Colitis and Irritable Bowel Disease

The inflammation curbing attribute(s) of IL-37 has been explored by employing IL-37tg mice in various studies [78]. The dynamics of IL-37 in dysregulated intestinal immune response (to bacterial antigens) typically featured in irritable bowel disease was investigated [91]. Clinical scores and histological indices in IL-37 expressing IL-37tg mice treated with dextran sulphate sodium (a colitis-inducing agent) were observed to be significantly reduced in comparison to IL-37 deficient controls, albeit transfection of bone marrow cells from hIL-37tg mice to wild-type age and sex matched litter mates resulted in protection from dextran sulphate sodium (DSS) induced colitis [91]. IL-37 typically exerts minimal effects on the secretion of IL-10 although IL-37 was speculated to mediate amplification of the anti-inflammatory actions of TGF-β owing to its association with a key intracellular effector of TGF-β signaling viz SMAD3 [78,91].

IL-37 is amplified in the inflamed mucosa of irritable bowel disease sufferers where it is speculated to be secreted in response to TNF-α triggered by nuclear factor (NF)-κβ in addition to activator protein stimulation. The anti-inflammatory role of IL-37 via negative-feedback action was demonstrated by an IL-37b mediated inhibition of TNF-α stimulated IFN-γ-inducible protein-10 release in the colonic sub epithelial myofibroblast consequently exerting an anti-inflammatory role [83].

### 4.2. A Role for IL-37 in Asthma

Hu et al. [92] illustrated a reduction in *IL-37* mRNA and a concomitant increase in the levels of IL-1β, IL-6 as well as TNF-α in LPS-stimulated sputum taken from asthmatic patients, the inflammatory cytokine expression observed was potently antagonized by co-incubation with IL-37 [92]. Stimulation of PBMC isolated from asthmatic patients elaborated lower levels of IL-37. Further investigations revealed reduced levels of key immune-mediating proteins (i.e., proline-serine-threonine phosphatase interacting protein-2, triggering receptor expressed myeloid cells) in asthmatic subjects compared to non-asthmatic controls. A study conducted in mice revealed a complete abrogation of the hallmarks of asthma (viz goblet cell hyperplasia and allergic airway inflammation) as well as decreased T_H_2 cytokine expression upon rIL-37 administration to OVA-sensitized mice. IL-37 has been documented to exert its immune-regulatory action via IL-18R and SIGIRR/IL-1R8.This protective effect of IL-37 was noticeably absent in mice lacking the SIGIRR/IL-1R8 receptor as well as in phosphate buffered saline (PBS)-treated mice [92].

### 4.3. Immunomodulatory Action of IL-37 in Collagen-Induced Arthritis

In collagen induced arthritic mice, local intra-articular injection of rIL-37 resulted in significantly delayed disease onset in addition to amelioration of arthritis-related clinical symptoms attributable to an IL-37 influenced downturn in the secretion of IL-17 and T_H_17 triggering cytokines, particularly IL-1β and IL-6 [93].

Adipose tissue inflammation, a major determinant of insulin resistance in chronically obese patients was abrogated in IL-37tg mice maintained on high fat diet [94]. Cavalli et al. [95] reported a role for IL-37 in inflammation-induced fatigue. They reported a progressive improvement in exercise tolerance (rota-rod test) to near baseline levels in mice treated with rIL-37 following systemic administration of LPS. Exercise tolerance in rIL-37 treated mice surpassed those seen in the rIL-1Rα-antagonist treated group as well as their saline treated counterparts. Such IL-37 mediated improved exercise tolerance was surmised to occur via IL-37 mediated stimulation of oxidative phosphorylation with an associated plunge in oxidative stress-related metabolites in addition to enhanced muscular mitochondrial respiration [95].

### 4.4. IL-37 in Cancer

Variable expression profiles of IL-37 have been demonstrated in a range of malignancies. Intra-tumoral administration of human *IL-37* gene in a murine model of fibro sarcoma promoted regression of intra-dermally established tumors. Furthermore, similarly administered multiple doses of *IL-37* gene completely halted tumor development. Moreover, IL-37 treatment conferred a cancer-specific immunity to previously susceptible mice upon treatment with rIL-37 such that they failed to succumb to a re-challenge despite increases in inoculum size of active malignant cells [96].

Abulkhir et al. [77] surmised that the proliferation and invasiveness of human papilloma virus (HPV) a recognized chronic inflammation-causing virus (implicated in cervical cancer pathogenesis) was potently inhibited by gene transfection with *IL-37* attributed to the reduction in expression and phosphorylation of STAT3 (an otherwise potent inducer of cell proliferation and invasion).

Zhao et al. [97] reported a negative correlation between tumor size and IL-37 expression in patients suffering from hepatocellular carcinoma. The study recognized that a greater IL-37 expression within the tumor microenvironment had a strong positive correlation and better prognosis translated in terms of increased overall survival (OS) as well as disease free survival [97].

In mouse models of breast carcinoma, IL-37 decreased tumor growth in immunocompetent mice. Although its effect was deduced to occur as a result of its regulatory influence on the tumor microenvironment rather than a targeted action on the malignant cells. Moreover, IL-37 was unable to afford an increased survival rate to mice afflicted with breast cancer cells presumably because a highly metastatic cancer cell line (4TI) was employed. Furthermore, IL-37 mediated tumor suppressing ability may be confined to inhibition of tumor proliferation through its action on CD4^+^ activation without having effects on tumor metastasis [98].

The regulatory potential of IL-37 on signal pathway activation of metastatic tumor cell lines was explored by Luo et al. (2017). Of the five known splice variants of IL-37, tumor (cell and tissue) levels of IL-37b isoform were consistently elevated and demonstrated an appreciable positive prognostic potential with disease progression in cancer patients. Elevations in *IL-37b* mRNA levels were positively correlated with overall survival whereas low expression levels were linked to poor patient prognosis. Additionally, upregulation of intracellular IL-37b significantly reduced the activation of various metastatic gene promoting pathways viz extracellular signal-regulated kinase (ERK), p38 mitogen-activated protein kinase (p38MAPK) , jun NH_2_-terminal kinase (JNK), phosphoinositide 3-kinase (P13K), STAT3 and NF-κB in addition to a potent reduction in the expression of molecules classically implicated in the promotion of tumor cell proliferation, invasiveness and viability (i.e., MMP-9, IL-6 and IL-8), conversely, these effects were completely abrogated upon downregulation of IL-37b expression [99]. Inactivating mutations in TGF-β/SMAD signaling pathway (particularly SMAD4 and to a lesser extent SMAD2) and by extension their cellular regulatory mechanisms have been implicated in various types of cancer [100]. Interestingly, the binding of mature and precursor forms of IL-37b to SMAD3 disrupted the formation of the SMAD2/SMAD4/SMAD3 tripartite complex invariably hindering the nuclear translocation of SMAD2 and SMAD4. IL-37 was hence able to suppress the SMAD signaling pathway tumor cell proliferation and invasiveness via competitive binding to SMAD3 [99]. Essentially, the action of intracellular IL-37b on the expression of various protein tyrosine phosphatase molecules was proffered as the probable mechanism of IL-37 mediated suppression of signal pathways and metastatic capacity of the tumor cell lines studied seeing as the inhibitory actions of IL-37b on tumor cell signaling pathways and metastasis were abolished upon incubation with pervanadate (a tyrosine phosphatase inhibitor). Suffice to say that intracellular IL-37b represents a novel biomolecule involved in negative regulation of the metastatic capacity and signal pathway of tumor cells and offers translational potential as a prognostic marker and therapeutic target for cancer management [99].

Further tumor-limiting roles have been linked to IL-37 in renal carcinoma and non-small cell lung carcinoma. IL-37 has been shown to correlate positively with disease severity in endometriosis where *IL-37* mRNA correlated inversely to NF-κβ [101]. Several mechanisms have been proffered to explain the varied actions of IL-37 in many types of cancers including; coordinated chemotaxis of immune cells, inhibition of STAT3 activation, cell migration and angiogenesis as well as promoting pseudo starvation to counteract the “Warburg effect” (aerobic glycolysis) of cancer cells [77].

### 4.5. Function of IL-37 in Cardiovascular Disorders

Coronary artery calcification (CAC) is a documented risk factor for adverse cardiovascular events [102]. Chronic inflammation has been identified as being contributory to the initiation and advancement of soft tissue calcification (viz coronary artery calcification). Cells of the immune system (lymphocytes, dendritic cells, etc.) permeate sclerotic plaques and activate the release of numerous cytokines which subsequently modulate calcification in arterial parenchyma [103]. Inflammatory cytokines participate in arterial wall calcification by activating and promoting differentiation of vascular smooth muscle cell into osteoblasts. (viz osteogenic differentiation). At variance with inflammatory cytokines, anti-inflammatory cytokines have been observed to ameliorate vascular calcification [103].

Arterial wall rigidity (stiffening) together with the accumulation of cellular components, cholesterol and associated extracellular matrices (i.e., plaques) constitute the defining feature of atherosclerosis. A direct relationship exists between the degree of CAC and atherosclerotic plaque burden [104]. Atherosclerosis is perceived to occur as a result of an interplay between elevated lipid content and inflammatory cytokine-driven endothelial dysfunction, several athrogenic features have so far been reported to be ameliorated by IL-37. A key modality in IL-37 mediated amelioration of athrogenic symptoms hinges on its action on macrophages. Athrogenic derived lipid-laden macrophages (i.e., foam cells) have been documented to release IL-37 hinting at the involvement of IL-37 in macrophage activation and their subsequent phenotypic shift to foam cells [85]. IL-37 is proposed to resolve atherosclerosis by modulating macrophage polarity and decreasing the effective size of an aortic plaque in relation to surrounding vascular cavity. Further experiments studying the dynamics of IL-37 murine arthrogenic subjects illustrated the ability of IL-37 to reduce plaque size and curb inflammatory cytokine driven calcification [85,104].

Chai et al. [105] observed decreased atherosclerotic area and plaque instability upon treatment of ApoE^−^/^−^ diabetic mice with a rIL-37 protein. The reduced calcification in murine subjects was evidenced by decreased tissue surface area showing positive stains for alkaline phosphatase (ALP) and bone morphogenic protein-2 (BMP-2) [105]. IL-37 was reported to ameliorate symptoms of atherosclerosis and arterial calcification via its influence on BMP-2 mediated ALP expression. BMP-2 is a well-known molecule which initiates osteoblast differentiation and atherosclerotic vascular calcification. Upregulation of IL-10 expression with a concomitant decrease in the production of TNF-α and IL-18 was put forward as a direct anti-inflammatory mechanism of IL-37 mediated amelioration of atherosclerosis and CAC. The beneficial effects of IL-37 in murine ApoE^−^/^−^ subjects was partially abolished upon concomitant administration of an antibody against the osteoclastogenesis inhibitory factor (anti-OCIF) viz anti-osteoprotegerin. Earlier in the study, elevated osteoprotegerin (OPG) titers were observed in mice treated with rIL-37, hinting at its probable involvement in fulfilling the beneficial actions of IL-37 in the disease model. As speculated, anti-OPG administration partially reversed the beneficial effects of IL-37 on plaque area and vulnerability, increased ALP and BMP-2 levels but failed to alter participating cytokine titers (i.e., IL-10, IL-18 and TNF-α), suggesting the involvement of yet another mechanism (independent of OPG) via which IL-37 mediates atherosclerosis and CAC amelioration [105].

A study by Xu et al. [106] noted that the toxic effects of inflammatory mediators on myocardial cells are surmounted by IL-37. Myocardial infarction a similarly debilitating cardiovascular disorder like atherosclerosis is equally ameliorated by IL-37 [106].

### 4.6. IL-37 and Its Role in Sleep-Wake Cycle

The participation of cytokines in sleep regulation has been widely recognized. Cytokines are especially cited as fundamental putative sleep factors that participate in regulation of the sleep-wake cycle. Normally released as acute phase proteins during immune activation (following perturbation by infection, inflammation or tissue damage), cytokines have been documented for their physiological role in sleep responses [107]. Krueger et al. [107] revealed the ability of systemically administered IL-1 to mimic symptoms of sleep deprivation (viz fatigue, performance impairment, poor memory and cognition, sleepiness, increased sensitivity to pain, etc.). A similar effect has been reported for TNF-α, another endogenous pyrogen, which at low doses was found to elaborate sleep-promoting effect in in vitro co-cultures of neurons and glia cells [108]. Variations in the cytokine repertoire during acute phase response to infections, inflammation or other similar immune perturbations have been reported to significantly alter sleep and morbidity responses [107,108].

IL-37 has been reported to antagonize the actions of inflammatory cytokines on host sleep-wake cycle following inflammatory treatment (i.e., LPS, rIL-1β and mouse influenza virus) by downregulating pro-inflammatory cytokine production while having no influence on levels of anti-inflammatory cytokines. IL-37tg expressing mice were reported to exhibit enhanced sleep responses and improved sleep indices as compared to their wild-type counterparts. Additionally, an overall reduction in mortality (increased survival, return to basal temperature and lesser weight loss) was reported for IL-37tg mice [109].

### 4.7. Immunological Significance of IL-37 in Leprosy

Leprosy represents a chronic mycobacterial illness in which disease progression is largely influenced by the disposition of the host’s immune response to the invading mycobacteria. The protracted (spectral) nature of this illness contributes to studies of the host’s innate immune responses pattern to an infectious pathogen. The disease offers an appealing model for investigating the regulation of immune responses to infection including the characterization of pathogen-associated molecular patterns, the dynamics of the cytokine milieu, as well as varying effector pathways during the course of an infection [110].

Tuberculoid leprosy (a clinical variant of leprosy infection) has been shown to evoke a powerful cellular mediated immune response in addition to significant delayed type hypersensitivity reaction to lepromin (an attenuated form of the leprosy bacillus), conversely a totally different immune scenario obtains in lepromatous leprosy (the other clinical spectrum of this illness). Apparently, in lepromatous leprosy a complete absence of specific cellular immune response as well as delayed type hypersensitivity response to the bacillus occurs. It is for this reason leprosy stands out for its tenacity in providing appreciable insights into the dynamics associated with immunological responses in host-pathogen relationships. Immune activation via the bipartite receptor complex TLR2/1 on dermal macrophages during tuberculoid leprosy mediate killing of the bacillus by promoting both T_H_1 and T_H_17 cytokine responses (IFN-γ, IL-2, TNF-α etc. which limit pathogen replication) while T_H_17 derived cytokines simultaneously predispose the host to tissue inflammation, macrophage activation, neutrophil recruitment as well as enhanced T_H_1 cell signaling which may result in reversal reactions (RR) or erythema nodosum leprosum (ENL). Contrarily, cytokine responses in lepromatous leprosy are mediated by the inflammation-mitigating cytokines (IL-4, IL-10 and a member of the leukocyte immunoglobulin-like subfamily of receptors dubbed LILRA-2) which inhibit TLR2/1 induce pro-inflammatory cytokine response and promote IL-10 release [111].

A study investigating the behavior of IL-37 during the course and clinical spectrum of leprotic syndromes (viz lepromatous leprosy, tuberculoid leprosy and their immunologically erratic borderline forms) revealed the ingenuity in the response of IL-37 to varying leprotic syndromes mediated through its action on macrophages, lymphocytes, endothelial cells as well as keratinocytes. IL-37 was shown to be significantly increased in macrophages and lymphocytes of dermal leprotic lesions as revealed by immunohistochemical characterization [111]. When compared to control tissues, lepromatous leprosy revealed greater IL-37 expression levels in macrophages in contrast to the tuberculoid form where elevations in IL-37 expression were confined predominantly to lymphocytes [111]. Nevertheless, overall elevations in IL-37 levels corroborate previous reports documenting elevation of IL-37 in relation to increasing amounts of inflammatory markers in chronic inflammatory processes such as psoriasis. Taken together, IL-37 is surmised to participate in the control of cellular immune response to *Mycobacterium leprae*, potentially mediating its suppressive function via its action on down-regulating TNF-α, IL-1β and T_H_17 signaling, modulating T_reg_ cell activity and promoting IL-10, Foxp3^+^ and TGF-β1 signaling [112]. The overall involvement of IL-37 in disease pathogenesis is summarized in Table 2 as below.

## 5. Conclusions

Outcome from independent experimentation on both healthy and diseased human, murine and (relevant) cultured cell populations have consistently corroborated the immune modulating potential of IL-35 and IL-37 in disease pathogenesis. The diverse actions of these novel cytokines (IL-35 and IL-37) range from their potential as biological markers of disease (severity) or progression to their direct beneficial action in amelioration of key disease features viz biochemical (pathogenic) mechanisms as well as modulation of acute phase responses (away from overt pathology/inflammation). In light of the promising immunomodulatory as well as anti-inflammatory actions elaborated by both IL-35 and IL-37 in a range of human diseases, these two cytokines represent potential enduring targets which can be modified for the advancement of highly efficacious therapies aimed at resolving (or managing) a range of debilitating human diseases. The existence of polymorphic variants of each cytokine observed to confer varying degrees of disease susceptibility or protection represents yet another research focus which can be exploited (blocked or enhanced) after extensive characterization for their biological roles in health and disease. Considering the endless possibilities that abound by their application in a wide variety of morbidities (or unique co-morbid states), the full impact of these cytokines hint at the possibility of novel discoveries in delineating disease pathways, drug/disease interactions as well as relevant much needed research and development.

## Figures and Tables

**Table 1:** Various roles of IL-35 in disease pathogenesis.

No.	Disorder	Biological Action	References
1.	Rheumatoid arthritis	↑ Promotes T_reg_ expansion	[6,35,38]
		↑ T_eff_ cell suppression	
		↓ T_H_1 differentiation	
		↓ T_H_17 differentiation	
		↑ IL-10 production	
		↓ angiogenesis	
2.	Experimental autoimmune encephalomyelitis	↑ IL-10 producing B_regs_	[32,33]
		↑ IL-35^+^ B_regs_	
		↓ IFN-γ expression	
		↓ IL-17 expression	
3.	Experimental autoimmune uveitis	↑ IL-35^+^ B_reg_ expansion	[32]
		↑ iTr35 expansion	
		Inhibition of pathogenic T_H_17 cells differentiation	
		Inhibition of pathogenic T_H_1 cells differentiation	
4.	Hashimoto’s thyroiditis	↑ T_reg_ proliferation (↑ immune tolerance)	[44]
		↓ thyroid cell apoptosis/auto destruction of thyroid tissue	
		↓ TPoAb producing B cells	
5.	Multiple low dose streptozotocin	↑ IL-35 expression	[45]
		↑ IL-10 expression	
		↑ Suppressive potential of T_regs_	
6.	Asthma	↓ airway CCL11 and CCL24 recruitment	[49]
		↓ IL-6 and T_H_1 cytokine expression	
		↓ STAT1 and STAT3 phosphorylation	
7.	Allergic rhinitis	↑ T_reg_ expansion	[51]
		↑ IL-10 expression	
		↓ T_eff_ cell proliferation	
		↓ T_H_2 cytokine expression	
8.	Cardiovascular diseases	rs2243115 and rs428253 linked (↓) decrease in type-2 diabetes mellitus and metabolic syndrome	[54]
9.	Systemic lupus erythematosus	↓ T_H_17 differentiation	[61]
		↑ peripheral and thymic IL-10^+^ B_regs_	
10.	Cancer	↓ CTL responses	[28,64,65,67,68]
		↑ recruitment of CD4^+^ CD25^+^ T_reg_	
		↑ gp130 expression by tumor cells	

(↑): Upregulation; (↓): Downregulation; STAT: Signal transducer and activator of transcription; CCL11: Eotaxin-1; CCL24: Eotaxin-2; TPoAb: Thyroid peroxidase antibody.

**Table 2:** Summary of the role of IL-37 in disease pathogenesis.

No	Disorder	Actions of IL-37	References
1.	Experimental colitis	↓ TNF-α expression	[91]
		↓ IL-1β expression	
		↓ Leukocyte recruitment	
		↑ Amplification of anti-inflammatory responses mediated by TGF-β	
2.	Asthma	↓ T_H_1 cytokine production	[92]
		↓ IL-17 production	
		↓ T_H_2 cytokine expression	
3.	Collagen induced arthritis	↓ T_H_17 cell proliferation	[93]
		↓ IL-17 triggering cytokines (viz IL-6, IL-1β, etc.)	
		↑ tolerogenic DCs (↓ DC-MAPK signal transduction)	
4.	Cancer	↓ tumor cell invasiveness and metastases	[77,96,99]
		↓ Angiogenesis	
		↓ IL-6, IL-8 and MMP-9	
		↑ NK cell recruitment	
		↑ PTP expression	
		↑ STAT3 inactivation	
5.	Cardiovascular disorders	↓ IL-18 expression	[85,104,105,106]
		↓ TNF-α expression	
		↓ BMP-2 and ALP expression	
		↑ IL-10 production	
		↑ OPG titers	
6.	Leprosy	↓ TNF and IL-1β expression	[110,111,112]
		↓ T_H_17 cell differentiation	
		↑ T_reg_ proliferation	
		↑ IL-10 expression	
		↑ TGF-β1 signaling	
7.	Sleep	Attenuation of pro-inflammatory cytokine mediated sleep deprivation	[109]

(↑): Upregulation; (↓): Downregulation; NK cell: Natural killer cell; BMP-2: Bone morphogenic protein-2; ALP: Alkaline phosphatase; STAT: Signal transducer and activator of transcription; MAPK: Mitogen activated protein kinase; DC: Dendritic cell; OPG: Osteoprotegerin.
